# Trends in total cholesterol screening and in prescribing lipid-lowering drugs in general practice in the period 1994–2003

**DOI:** 10.1186/1471-2296-9-39

**Published:** 2008-06-30

**Authors:** Stefaan Bartholomeeusen, Jan P Vandenbroucke, Carla Truyers, Frank Buntinx

**Affiliations:** 1 Department of General Practice, Katholieke Universiteit Leuven, Kapucijnenvoer 33, Leuven, Belgium; 2 Clinical Epidemiology, Rijksuniversiteit Leiden, Leiden University Medical Centre, Leiden, The Netherlands; 3 Department of General Practice, Katholieke Universiteit Leuven, Kapucijnenvoer 33, Leuven, Belgium; 4 Research Institute Caphri, Universiteit Maastricht, The Netherlands

## Abstract

**Background:**

General Practitioners (GPs) play a central role in controlling an important risk factor for cardiovascular diseases, i.e. cholesterol levels in serum. In the past few decades different studies have been published on the effect of treating hyperlipidemia with statins. Guidelines for treatment have been adopted. We investigated the consequences on the practice of GPs screening cholesterol levels and on the timing of starting statin prescription.

**Methods:**

For this descriptive study, data from the Intego database were used, composed with data from the electronic medical records (EMR) of 47 general practices in Flanders. GPs had not received special instructions for testing specific patients. For each patient the mean cholesterol level per year was calculated. A patient belonged to the group with lipid-lowering drugs if there was at least one prescription of the drug in a year in his EMR. Mixed model linear regression models were used to quantify the effect of covariates on total cholesterol values.

**Results:**

In the period 1994–2003 total cholesterol was tested in 47,254 out of 139,148 different patients. Twelve percent of those tested took lipid-lowering medication. The proportion of patients with at least one cholesterol test a year, increased over a period of ten years in all age groups, but primarily for those over the age of 65.

The mean cholesterol level decreased in the treated as well as in the non-treated group. Of the patients with a cardiovascular antecedent who were on lipid-lowering drugs in 2003, 56% had a cholesterol level ≤ 199 mg/dl, 31% between 200–239 and 13% over 240 mg/dl.

**Conclusion:**

The indications for testing and treating cholesterol levels broadened considerably in the period examined. In 2003 cholesterol was tested in many more patients and patients were already treated at lower cholesterol values than in previous years. Comparisons of cholesterol levels over different years should therefore be interpreted with caution as they are a reflection of changes in medical care, and not necessarily of efficacy of treatment.

## Background

For the primary and secondary prevention of cardiovascular diseases, it is important that the General Practitioner (GP) knows the cardiovascular risk factors of his/her patients and, if necessary, reduces the risk through an intervention. Primary care is the preferred setting for improvement of the risk factors, because the GP is the patient's first and most prominent contact with health care [1,2].

Over the last few decades death rates from coronary heart disease have decreased by 50% in the United States, Europe and Australia [3]. Despite this, heart disease remains the greatest cause of death in industrialized countries. It is possible to control a number of major risk factors: smoking, hypercholesterolaemia, hypertension, glucose intolerance and obesity. Hypercholesterolemia is one of the major risk factors for cardiovascular diseases [4] and the positive effect of lowering cholesterol levels in the serum has been proved in several studies [5-7]. The effect of lowering cholesterol levels on primary prevention for cardiovascular diseases was pointed out in a study of patients with an increased cholesterol, namely 272 mg/dl at the start of the study [6]. Recently it has been suggested that the control of three risk factors in primary prevention, i.e. smoking, total cholesterol and blood pressure, would be responsible for half of the decrease in mortality for coronary diseases in England and Wales [8,9].

What is interesting is that high cholesterol values can be controlled relatively easily by the use of lipid-lowering medication in most cases. Moreover, it would take only a minor decrease in cholesterol levels in the whole population to have a major effect on mortality, in contrast with highly sophisticated interventions and treatments, which merely have a positive effect on mortality in a restricted number of people [10,11]. In Belgium 90% [12] and in Denmark 82% [13] of the lipid-lowering medication is started by the GP. The GP can play an important role in recognizing the cardiovascular risk factors of his/her patients. After all, they treat patients for a whole range of diseases, long before an infarction or cerebrovascular accident (CVA) occurs.

Using data from a database of EMRs of GPs in Flanders, we examined trends in testing cholesterol and prescribing of lipid-lowering drugs in general practice in the period 1994–2003 and also investigated whether the target of optimal treatment was reached in patients with and without cardiovascular risk factors. This study is based on information from a database containing data from the EMR of GPs.

## Methods

### Design

This is a descriptive study, based on data from the Intego database.

### The Intego database [14,15]

The Intego database is composed of data from the EMRs of GPs using the medical software program Medidoc^®^. In the spring of 2004, data were collected from 47 general practices with 55 GPs, spread over Flanders. All diagnoses and medical prescriptions had been recorded by the GP using thesauri with specific codes. Laboratory results were sent from the laboratory to the general practice via a protected connection and were automatically imported with program-specific keywords into the EMR. General practices were selected on the basis of high quality of registration. The database contains information on diagnoses, drug prescriptions and laboratory results of 140,000 different patients. The population sample from the database is representative of the Flemish population on age, sex and geographical spread [16]. Epidemiological figures have been calculated from 1994 on. The number of different patients seen in a calendar year in a practice is the yearly contact group (YCG) and this figure is used as a denominator. Only for the patients in the YCG of 2003 was a calculation made as to how many of them had a cholesterol test in the previous five years.

### Measurements

The GPs were left to decide for themselves which patients would have their cholesterol levels tested. For this study GPs were not given specific directives for prescribing blood tests in specific patients. In this way, tests were obtained in patients at high risk but also in patients who consulted for other reasons. The total cholesterol level was examined by the local laboratory and not by one central laboratory. Because this study had not the intention to determine an exact cardiovascular risk profile, a complete lipid profile was not considered in this study.

### Analysis

The mean cholesterol value was calculated per patient, based on all the tests performed during a specific year in the period 1994–2003. For the conversion from mg/dl to mmol/l values are multiplied by 0.0259 and for the reverse by 38.61 [17]. Each patient was classified in the group treated with lipid-lowering medication in a specific year, if at least one prescription was recorded for this class of medication in the year concerned. If a prescription had been recorded in at least one year of the period 1994–2003, the patient was added to the group of treated patients.

Patients were included in the group with a cardiovascular antecedent, if at least one of the following codes of the International Classification of Primary Care (ICPC-2) appeared in their medical history: K74 Ischaemic heart disease with angina, K75 Acute myocardial infarction, K76 Ischaemic heart disease without angina, K89 Transient cerebral ischaemia, K90 Stroke/Cerebrovascular accident, K91 Cerebrovascular disease, K92 Atherosclerosis/peripheral vascular disease. The cut-off points for optimal treatment of the total cholesterol levels were based on the third report of the US National Cholesterol Education Program [18]. A fasting total cholesterol <200 mg/dl is good, between 200 and 239 is borderline high and ≥ 240 is too high.

### Statistics

Because of the large number of observations, the following formula was used for the standard error in the calculation of 95% confidence intervals around the difference in cholesterol values,: $\sqrt{\frac{s_{1}^{2}}{n_{1}}+\frac{s_{2}^{2}}{n_{2}}}$, where *s*_1_and *s*_2 _are the standard deviations and *n*_1 _and *n*_2 _the number of patients in the two groups. The z test [19] was used to test statistical significance for the difference in cholesterol values.

Mixed model linear regression models incorporating an unstructured covariance structure, accounting for repeated measures within subjects and random effects at the practice level, were used to quantify the effect of the covariates on the cholesterol values: fixed effects were defined as treatment effect (defined as having ever been prescribed lipid-lowering drugs), sex, age, time and the interaction between time and treatment. These analyses were performed using SAS software, version 9.1.

## Results

### Patients with a cholesterol test

In the period 1994–2003 the total cholesterol level was tested at least once in 47,254 out of 139,148 patients (54.56% women and 45.44% men). In those patients 131,000 cholesterol levels were calculated. Because of the small number of tests administered to patients under age 24, only the patients over the age of 25 will be discussed, i.e. 125,342 values and 43,197 different patients. Of all the patients seen in the practice in 2003, 57% had their cholesterol tested at least once in the preceding five years. The proportion of patients with at least one cholesterol test in the year 2003 in the age groups 25–44, 45–64, 65–74 and 75+ amounts to 16%, 38%, 56% and 42% respectively of the patients who had contacted the practice in that year (Table 1). This is an increase compared with the year 1994 of 4%, 8%, 16% and 14% respectively.

**Table 1:** Proportion of patients with a cholesterol test and proportion with a cholesterol test and with lipid-lowering drugs, in the years 1994 and 2003

age group	proportion of patients in YCG* with cholesterol test				proportion of patients with medication in the group with cholesterol test (%)	
	
	1994		2003		1994	2003
	n	%	n	%		
25–44	2304	12.20	2924	15.80	8.72	5.54
45–64	3927	29.69	6493	37.91	32.82	26.17
65–74	2097	39.68	3705	55.91	33.14	40.89
75+	925	27.78	2672	41.51	11.35	35.40

*yearly contact group (patients seen in the practice during a year)

In the group with a cholesterol test, 5,768 patients (12.21%) took a lipid-lowering drug, and the proportion of patients taking medication in the age groups 25–44, 45–64, 65–74 and 75+, amounts to 6%, 26%, 41% and 35%, respectively, in the year 2003. In the period 1994–2003 the proportion of patients taking lipid-lowering drugs increased in the age group 65+ years and decreased under this age (Table 1).

### Patients on a lipid-lowering drug

The increase in the number of patients with a prescription for a lipid-lowering drug resulted from the increasing use of statins and not of fibrates. The proportion of patients of the YCG, with at least one prescription for a statin in a specific year, increased from 0.47 in 1994 to 5.30% in 2003. The increase was noticeable in all age groups particularly after 1997. In the same period, there was only a slight increase in the use of fibrates from 1.45 to 1.81%.

**Figure 1:**
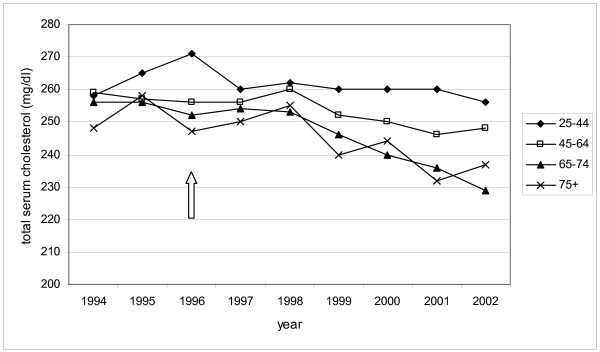
Mean total cholesterol levels in different age groups in the year before lipid-lowering drugs were started, in the period 1994–2002; arrow indicates facilitation of the reimbursement of lipid-lowering drugs.

In 2003, GPs started their patients, specially the oldest age group, on lipid-lowering drugs at lower cholesterol values, compared to 1994. In 1994 there was no great difference in cholesterol levels between the age groups according to the cholesterol level at which lipid-lowering drugs were started. In 2002 the levels were much lower in the oldest age group than in the younger age groups (Figure 1).

In patients who had taken a cholesterol test, the proportion with an additional cardiovascular risk factor (history of cardiovascular disease, hypertension, diabetes) increased from 29% to 34% between the years 1994–1995 and 2002–2003. In this last group of patients with a risk factor, the proportion of patients treated with a lipid-lowering drug increased from 14% to 29%.

### Cholesterol values

The results of the linear mixed model suggest that on average cholesterol levels were lower for patients that were not on lipid-lowering drugs. With increasing age the total cholesterol also increased. Females had a tendency to have higher levels of total cholesterol in comparison to males, p < 0.0001 (Table 2).

**Table 2:** Fixed effects for the final model for total serum cholesterol

Effect	Estimate	Standard Error	DF	t Value	Pr > |t|
Intercept	15079	294.18	49	51.26	<.0001
Treatment (no)	-11179	344.77	88E3	-32.42	<.0001
Age	0.4457	0.008216	88E3	54.25	<.0001
Sex (female)	5.3299	0.2603	88E3	20.48	<.0001
Time	-1.8572	0.09101	88E3	-20.41	<.0001
Treatment*Time	-5.5843	0.1723	88E3	-32.40	<.0001

**Figure 2:**
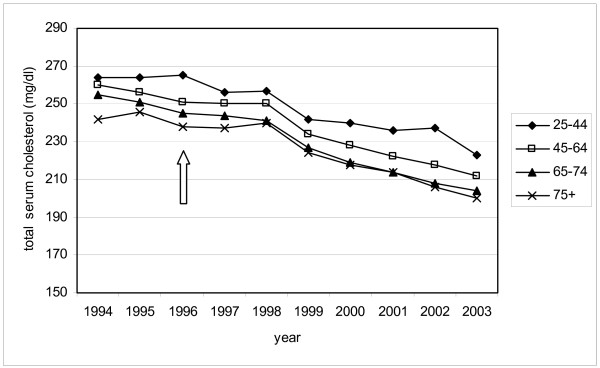
Mean total cholesterol levels in patients with lipid-lowering drugs in different age groups, in the period 1994–2003; arrow indicates facilitation of the reimbursement of lipid-lowering drugs.

**Figure 3:**
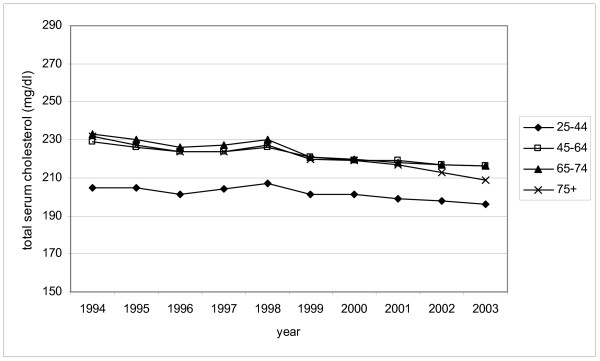
Mean total cholesterol levels in patients without lipid-lowering drugs, in different age groups in the period 1994–2003.

The mean total cholesterol level decreased significantly in all patients between 1994 and 2003, especially in patients taking lipid-lowering drugs. The range of decline in the different age groups among patients taking lipid-lowering medication was 10 mg/dl (from 41 to 51 mg/dl) (Table 3). The decrease appeared after 1999 in particular, three years after the facilitation of the reimbursement of the statins, which resulted in an increase in prescriptions of these drugs (Figure 2). In the group without lipid-lowering drugs we observed a similar decrease, but its magnitude reached only about a third compared to the group with medication (Table 3, Figure 3).

**Table 3:** Mean total cholesterol values (SD) and difference between the year 1994 and 2003 (95%CI) in patients with and without lipid-lowering drugs

Age	1994		2003		difference	
			
	medication		medication		medication	
	without	with	without	with	without	with
25–44	205 (38.07)	264 (41.51)	196 (38.50)	223 (48.78)	9 (6.83–11.17)*	41 (31.68–50.32)*
45–64	229 (40.58)	260 (40.68)	216 (36.28)	212 (40.82)	13 (11.14–14.86)*	48 (45.05–50.95)*
65–74	233 (40.78)	255 (41.78)	216 (35.40)	204 (37.35)	17 (14.40–19.60)*	51 (47.51–54.49)*
75+	232 (45.95)	242 (42.73)	209 (39.79)	200 (38.34)	23 (19.34–26.66)*	42 (47.51–54.49)*

*p < 0.0001

The covariance estimates suggest that practices do not differ in their average cholesterol scores and that most variation occurs among patients within practices. (The variance component within practices is more than 250 times larger than the size of the variance component between practices).

### Targets

Seventeen percent of the treated patients still had a total serum cholesterol level which was considered to be elevated, 47% were well regulated and 36% were borderline. The proportion of patients treated with lipid-lowering drugs in 2003, who had a previous history of a cardiovascular disease and who reached the target of ≤ 199 mg/dl was 56%. Only 43% of patients without such a disease reached the target cholesterol level.

## Discussion

### Summary of main findings

In a relatively short period, the indication for testing and drug treatment of elevated cholesterol levels as a cardiovascular risk factor has broadened greatly. Cholesterol testing was performed in more patients and lipid-lowering drugs were prescribed earlier. This was the case in all age groups but especially in the over-45 years old group. This has consequences for the mean values of total cholesterol and, for this reason, comparisons of cholesterol levels in the different years should be interpreted with caution. The target of a cholesterol value below 199 mg/dl was only reached in 47% of the patients treated in 2003.

Since the GP has access to the information on the cholesterol level of approximately more than half of his/her patients over the age of 25, he/she characterize the cardiovascular risk profile of these patients by asking them some additional questions. In this way the GP can play a more important role in the primary and secondary prevention of cardiovascular diseases.

The fact that GPs tested cholesterol levels at an earlier time and prescribed lipid-lowering drugs sooner is probably the result of the clinical studies that have shown the positive effects of lowering high cholesterol levels and of the different guidelines that have been published in literature. Advertising by the pharmacological industry and the facilitated reimbursement conditions of statins by the Belgian health care system could have also played a role encouraging the prescription of statins.

Shortly after the introduction of statins in 1991 they were only reimbursed by the Belgian Health Insurance after the patient had followed a diet and if the GP had established that the use of fibrates appeared to be ineffective. Only from 1996 on was reimbursement facilitated and were statins reimbursed if after three months of dieting the total cholesterol remained over 250 mg/dl.

It is not clear why patients above age 65 years old were tested more often and were also prescribed more frequently lipid-lowering drugs than younger patients. This was also found in another Belgian study [12]. One would expect a more aggressive approach in younger age groups as the benefits of lowering cholesterol would be larger [20]. It might be possible that the elderly consult a doctor more often for various reasons such as a larger number of associated comorbidities; and, because of this, blood tests were carried out more frequently.

The fact that serum cholesterol blood samples were examined by the local laboratory and not by one central laboratory is not considered a problem. One can assume that the quality of the laboratories is appropriate, because they are regularly subjected to internal and external quality controls, in order to reserve the right to have their activities reimbursed by the social security authorities. Although the tests were done in many different laboratories, retesting of patients was usually done in the same laboratory as the one in which the first test was carried out.

Remarkably, scarcely half of the treated patients reached the target of 199 mg/dl. Similar findings have been also confirmed by other authors [1,13,21]. It appears that doctors do not adapt their behaviour immediately to the current guidelines. This is probably also due to the fact that it is often difficult to motivate patients to take even more medication to further decrease their cholesterol levels.

The findings of this study suggest that the practice of GPs changed considerably in the period 1994–2003. Firstly, they prescribed more cholesterol tests, also in patients with less or no risk factors. Secondly, they already treated patients with lower initial total serum cholesterol values. Both these factors may have lead to a decrease in the average serum cholesterol levels that were measured. In addition, some of the change in cholesterol levels could be explained by the effect of regression to the mean. Thus, the overall positive evolution of the cholesterol levels found in this study, may in part result from these factors, and may not really reflect a lowering of the average serum cholesterol in the population.

### Strengths and the limitations of this study

The strength of this study is the fact that it originates from primary care data. The GPs were not informed that cholesterol levels would be examined and neither were they given instructions as to which patients had to be tested. Blood tests were performed for diverse reasons and without any selection of patients. These results give an idea of the "normal" working method of the GP. This method of data collection makes it possible to examine such large numbers of patients from different sex and age groups [22].

A weak point in this study is the fact that the GPs in this study are not a random sample of Flemish GPs. They were not selected because they worked in a more, or less, scientific way than their colleagues, but because of the high quality of their registration [14]. The patient population, however, is comparable with the population in Flanders regarding age and sex.

It is possible that more tests were performed than recorded, eg. by hospital consultants. This may have some influence on the cholesterol levels before starting drug treatment. In Belgium the GP is usually informed about test results prescribed by consultants in such a way that he is unable to include them in the laboratory module of their EMR. Unfortunately, a lipid profile was not available for analysis in this investigation.

Only in a small percentage of the GP files was the patients' smoking status formally recorded. It was therefore not possible to use our data to calculate the compliance with current guidelines on cardiovascular risk control.

### Comparison with literature

Our results agree with those of formerly published papers. Filippi found that in Italy in one year, a cholesterol test was carried out in 50% of patients in general practice above the age of 65 [23]. In a study that examined how recommendations were being followed in primary care, 60% of the patient samples had at least one total cholesterol measurement. Older patients had also undergone more cholesterol tests than younger patients [24]. Svilaas found a lower barrier to administer lipid-lowering drugs from 1995 onwards [21]. The increase in the prescribing practices of lipid lowering drugs has been described in several studies. Baxter describes an exponential increase from 1994 on [25]. According to information from the General Practice Research Database in 228 practices in England and Wales, there was more than a 10-fold increase in statins prescribing by GPs between 1991 and 1997 [26]. Of 114 patients with a history of coronary heart disease and taking lipid-lowering drugs, only 44% had a total cholesterol level below 190 mg/dl [27].

## Conclusion

It is possible that in this study population it was relatively easy for GPs to define a cardiovascular risk profile for their patients. They had access to the cholesterol levels of more than half of their patients over the age of 25. The risk profile can be further refined by means of additional questions. GPs have dramatically changed their attitude concerning the testing of cholesterol levels, but still fail to achieve the targets for optimal lipid-lowering treatment in approximately half of the treated patients. Therefore, it is advisable for professional education to concentrate first and foremost on the use of published guidelines among physicians prescribing in the primary care setting.

## Competing interests

The authors declare that they have no competing interests.

## Authors' contributions

SB conceptualised the idea for the study, did the literature review, analysed the data, interpreted the results and wrote the manuscript. JVDB participated in the analysis and interpretation of the results and revision of the manuscript. CT participated in the analysis of the results. FB participated in the analysis and interpretation of the results and the revision of the manuscript.

## Pre-publication history

The pre-publication history for this paper can be accessed here:


